# *Galgeun-tang* modulates lipid, glucose, and energy metabolism in diet-induced obesity across cellular, nematode, and murine models

**DOI:** 10.3389/fphar.2026.1747882

**Published:** 2026-02-27

**Authors:** Song-Yi Han, Seo-Hyun Park, Chanuk Heo, Hojun Kim

**Affiliations:** 1 Department of Rehabilitation Medicine of Korean Medicine, Dongguk University, Goyang, Republic of Korea; 2 Department of Rehabilitation Medicine of Korean Medicine, Dongguk University Bundang Oriental Hospital, Seongnam, Republic of Korea

**Keywords:** botanical drugs, *Caenorhabditis elegans*, energy metabolism, Galgeun-tang, glucose metabolism, lipid metabolism, obesity

## Abstract

**Background:**

*Galgeun-tang* (GGT) is a traditional Korean multi-component formulation composed of several botanical drugs and has long been prescribed for febrile and musculoskeletal disorders. With the global rise in obesity and obesity-related metabolic diseases, there is increasing demand for safer and multi-targeted therapeutic strategies. However, the systemic metabolic effects and anti-obesity potential of GGT remain incompletely understood.

**Methods:**

The anti-obesity effects of GGT were evaluated using a tiered experimental approach comprising C2C12 myotubes, high-fat diet (HFD)-induced obese C57BL/6J mice, and *Caenorhabditis elegans* (*C. elegans*) exposed to high-glucose conditions. *In vitro* analyses assessed glucose uptake, gene expression, and protein signaling pathways. In mice, body weight, glucose tolerance, serum biochemical parameters, histological changes, and hepatic and adipose gene expression were examined. In *C. elegans*, lifespan, lipid and glucose accumulation, and insulin signaling–related gene expression were analyzed following treatment with GGT or metformin (MET).

**Results:**

GGT enhanced glucose uptake and increased the expression of insulin-responsive and mitochondrial regulatory genes in C2C12 myotubes. In HFD-fed mice, GGT attenuated body weight gain, improved glucose tolerance and insulin sensitivity, and alleviated hepatic steatosis and adipose hypertrophy, accompanied by suppression of lipogenic genes and induction of β-oxidation markers. In *C. elegans*, GGT reduced lipid and glucose accumulation, prolonged lifespan, and modulated the expression of insulin signaling–related genes, including *daf-16* and *daf-2*. Across models, GGT exerted metabolic benefits in a dose- and context-dependent manner, with effects comparable to those of MET.

**Conclusion:**

GGT improves obesity-related metabolic dysfunction by coordinately regulating glucose homeostasis, lipid metabolism, and energy expenditure across cellular, nematode, and murine models. These findings provide preclinical evidence supporting GGT as a multi-targeted herbal intervention for obesity and metabolic disorders and warrant further targeted mechanistic studies and clinical investigations.

## 1 Introduction

Obesity has emerged as a major global health challenge, contributing to the increasing prevalence of metabolic disorders such as type 2 diabetes, cardiovascular disease, and non-alcoholic fatty liver disease (NAFLD) (Tan, 2004). It is characterized by dysregulated lipid storage, impaired glucose homeostasis, insulin resistance, and chronic low-grade inflammation (Busebee et al., 2023). In addition to these metabolic abnormalities, sedentariness and lack of physical activity are now recognized as major contributors to the development and progression of obesity, as they exacerbate energy imbalance and metabolic dysfunction (Magkos et al., 2020; Sahu et al., 2024). Although several pharmacological agents are currently available, conventional anti-obesity therapies often exhibit limited long-term efficacy or are associated with adverse effects, underscoring the need for safer and multi-targeted therapeutic strategies (Müller et al., 2022; Pati et al., 2023; Lu et al., 2025).

Traditional formulations are increasingly being explored as complementary approaches for metabolic regulation due to their pleiotropic biological activities. *Galgeun-tang* (GGT), also known as *gagen-tang* in Chinese and *kakkon-to* in Japanese, is a traditional Korean multi-component formulation composed of multiple botanical drugs, including *Pueraria montana* var. lobata (Puerariae Radix), *Ephedra sinica* (Ephedrae Herba), *Cinnamomum cassia* (Cinnamomi Ramulus), *Paeonia lactiflora* (Paeoniae Radix), *Glycyrrhiza uralensis* (Glycyrrhizae Radix), *Zingiber officinale* (Zingiberis Rhizoma), *and Ziziphus jujuba* (Zizyphi Fructus) (Song et al., 2020). Traditionally, GGT has been prescribed for the treatment of febrile illnesses associated with the common cold or influenza, inflammatory respiratory disorders, and musculoskeletal pain involving the head, neck, and shoulder regions, as documented in both experimental and clinical studies (Takayama et al., 2020; Ogawa-Ochiai et al., 2022; Katsuki et al., 2023; Takayama et al., 2023). Mechanistic investigations have further explored its pharmacological actions in inflammatory and infectious disease contexts (Zi-Kai et al., 2019).

In recent years, these traditional indications have prompted growing interest in the potential application of GGT to metabolic disorders, particularly those characterized by chronic inflammation and dysregulated energy homeostasis. Experimental studies have reported that GGT improves lipid profiles, modulates appetite-related gene expression, and alters gut microbiota composition in high-fat diet (HFD)-induced obese mice (Jeong et al., 2014). Comparable anti-obesity trends have also been reported in regional studies, including modulation of appetite-related gene expression and gut microbiota composition (Ki et al., 2016; Ye et al., 2016; Ko et al., 2020).

In addition, major plant-derived metabolites within GGT exhibit complementary metabolic activities. Puerarin, a principal isoflavone from *P. montana var.* lobata, has been shown to enhance insulin sensitivity, promote fatty acid oxidation, and inhibit adipocyte differentiation (Lee et al., 2010; Chen et al., 2018). Ephedrine and related alkaloids from *E. sinica* have been reported to increase thermogenesis, suppress appetite, and influence gut microbial composition (Kim et al., 2014; Yoo et al., 2021). Collectively, these findings suggest that GGT may exert multi-targeted regulatory effects on lipid, glucose, and energy metabolism.

Despite these promising observations, the systemic and dose-dependent metabolic actions of GGT across different biological levels remain insufficiently characterized. In the present study, we investigated the anti-obesity effects of GGT using a tiered experimental design. C2C12 myotubes were employed to assess cellular glucose uptake and metabolic gene regulation, while HFD-induced obese mice were utilized to validate systemic metabolic outcomes in a mammalian context. In addition, *Caenorhabditis elegans* (*C. elegans*) was incorporated as a supportive whole-organism model to examine whether the metabolic effects observed in cellular and mouse models are conserved at the organismal level under high-glucose conditions.

Through this integrative approach, the present study aims to provide preclinical evidence for the multi-targeted metabolic actions of GGT and to evaluate its potential as a complementary strategy for the management of obesity and metabolic dysfunction.

## 2 Materials and methods

### 2.1 *Galgeun-tang* (GGT) preparation and high-performance liquid chromatography -ultraviolet spectrometer (HPLC-UV) analysis

*Galgeun-tang* (GGT) granules were obtained from Hanpoong Pharm and Foods Co., Ltd. (Jeonju, Republic of Korea) that followed Good Manufacturing Practice (GMP) procedures. GGT is composed of seven botanical drugs, all of which are listed in the Korean Pharmacopoeia (KP).

All botanical drugs were authenticated by the manufacturer using morphological identification and pharmacopoeial criteria, and confirmed by Prof. Park, Sun-dong, a pharmacognosist at Dept. of Formula Science in Korean Medicine Dongguk University. Voucher specimens (Specimen Nos. GGT-001–002) have been deposited in the Herbarium of Dongguk University, Republic of Korea.

The botanical drugs were identified as: *P. montana var. lobata* (Willd.) Sanjappa and Pradeep [Fabaceae; Puerariae Radix], *E. sinica* Stapf [Ephedraceae; Ephedrae Herba], *Z. jujuba* Mill [Rhamnaceae; Zizyphi Fructus], *Neolitsea cassia* (L.). Kosterm. [Lauraceae; Cinnamomi Ramulus], *P. lactiflora* Pall. [Paeoniaceae; Paeoniae Radix], *G. uralensis* Fisch. [Fabaceae; Glycyrrhizae Radix], and *Z. officinale* Roscoe [Zingiberaceae; Zingiberis Rhizoma] (Supplementary Table S1).

The combined botanical drugs were extracted with 10 volumes (v/w) of distilled water at 100 °C for 3 h. The aqueous extract was then filtered and concentrated under vacuum below 60 °C, followed by drying to yield a powdered extract. The extraction yield was approximately 23.2%, corresponding to a drug–extract ratio (DER) of approximately 4.5:1 (w/w).

Quantitative analysis of ephedrine, glycyrrhizic acid, paeoniflorin, and puerarin was performed by HPLC-UV at Hanpoong Pharm & Foods, with compound-specific analytical methods optimized for each metabolite (Supplementary Table S2). The HPLC–UV analysis was conducted as a targeted quality-control method based on pharmacopoeial marker metabolites to ensure batch consistency of the GMP-manufactured extract, rather than as a comprehensive metabolomic fingerprint.

### 2.2 Cell culture and differentiation

Murine C2C12 myoblasts (American Type Culture Collection, ATCC, Manassas, VA, United States) were maintained in Dulbecco’s modified Eagle’s medium (DMEM; Welgene, Daegu, Republic of Korea) supplemented with 10% fetal bovine serum (Gibco, CA, United States) and 1% penicillin-streptomycin (Invitrogen, CA, United States) at 37 °C, 95% relative humidity, and 5% CO_2_. For differentiation, cells were cultured in DMEM containing 2% horse serum (Invitrogen) for 4–6 days until ≥90% of myoblasts had formed myotubes (Kislinger et al., 2005).

### 2.3 Cell viability assay

C2C12 myotubes were seeded in 96-well plates (1 × 10^5^ cells/well) and treated with GGT (0.4–2.0 mg/mL) for 24 h. Cell viability was determined using the EZ-Cytox assay kit (Daeil Lab Service, Seoul, Republic of Korea) according to the manufacturer’s instructions. Absorbance was measured at 450 nm with a microplate reader (Tecan Spark^Ⓡ^; Tecan Group Ltd., Männedorf, Switzerland). The viability of the control cells, in terms of absorbance, was set to 100%.

### 2.4 Glucose uptake assay in C2C12 myotubes

Glucose uptake was measured using 2-deoxy-2-[(7-nitro-2,1,3-benzoxadiazol-4-yl) amino]-d-glucose (2-NBDG; Invitrogen), a fluorescent derivative of glucose. Differentiated C2C12 myotubes were incubated in DMEM containing 2.5 mM glucose for 2 h, then treated with metformin (0.75 mM, Sigma-Aldrich, MO, United States) or GGT (1.0 mg/mL) for 12 h. Cells were exposed to 2-NBDG (50 μg/mL, Invitrogen) for 1 h, washed twice with Dulbecco’s phosphate-buffered saline (DPBS; Welgene), and fluorescence was measured at 485/535 nm using a microplate reader (Tecan Spark^Ⓡ^). Fluorescence images of C2C12 myotubes were examined under an epifluorescence optical microscope (BX53; Olympus, Tokyo, Japan) equipped with a digital camera device (DP73; Olympus) and a mercury lamp (U-RFL-T; Olympus). Images were captured using a ×10 objective lens. Image acquisition was performed using cellSens Standard software (version 1.7.1; Olympus) under identical exposure and gain settings for all experimental groups. The fluorescence intensity was quantified using ImageJ software (version 1.43; NIH, Bethesda, MD, United States).

### 2.5 Animal experiments

Forty-eight male C57BL/6J mice (6 weeks old, 18 ± 1 g; Daehan Biolink Co., Ltd., Eumseong, Republic of Korea) were acclimatized for 2 weeks under a 12 h light/dark cycle at a constant temperature of 25 °C and humidity levels of 50%–60%. The animals were provided with free access to a standard chow diet (38057; Purina Korea Inc., Seoul, Republic of Korea) and water. After acclimatization under specific pathogen-free (SPF) conditions, mice were randomly assigned to six groups (n = 8 per group): normal diet (ND), high-fat diet (HFD), metformin (MET; 100 mg/kg), and GGT at low (GGT-L; 504 mg/kg), medium (GGT-M; 612 mg/kg), or high (GGT-H; 720 mg/kg) doses.

The high dose of GGT (720 mg/kg) was determined by converting the clinically prescribed human dose to a mouse-equivalent dose based on body surface area normalization. The conversion was performed according to the FDA-recommended dose translation guidelines (Nair et al., 2018), using a clinical human dose of 12 mg/kg/day. The medium and low doses were set at approximately 85% (612 mg/kg) and 70% (504 mg/kg) of the high dose, respectively, to evaluate dose-dependent effects within a clinically relevant range.

Oral administration of distilled water (vehicle), MET, or GGT was initiated immediately after group allocation and continued once daily by oral gavage for a total experimental period of 10 weeks. During the initial 2-week pretreatment phase, all mice were maintained on a chow diet to allow stabilization of drug exposure before dietary challenge. This was followed by an 8-week dietary intervention phase, during which mice in the ND group were fed a normal diet (10% kcal fat; D12450B, Research Diets, NJ, United States) with regular drinking water, whereas mice in the HFD and treatment groups were fed a high-fat diet (45% kcal fat; D12451, Research Diets) supplemented with 10% fructose in drinking water.

The combination of a high-fat diet with fructose-supplemented drinking water was employed to exacerbate insulin resistance and metabolic dysfunction, as this dietary paradigm more closely mimics Western dietary patterns and has been widely used to induce obesity, glucose intolerance, and hepatic steatosis in rodents (Lim et al., 2010; Softic et al., 2019; Radhakrishnan et al., 2021).

This experimental design allowed assessment of the preventive and modulatory effects of GGT on the development of diet-induced obesity and metabolic dysfunction under high-fat/high-fructose conditions. Body weight was recorded weekly, and food intake was measured twice per week.

### 2.6 Oral glucose tolerance test (OGTT)

Following a 16 h overnight fast, mice were administered a sterilized glucose solution (2 g/kg; Sigma-Aldrich) via oral gavage. Blood glucose levels were measured from the tail vein at 0, 30, 60, 90, and 120 min using a handheld glucometer (Accu-Chek Active; Roche Diagnostics, Switzerland). The area under the curve (AUC) was calculated from glucose–time plots (Choi et al., 2021).

### 2.7 Serum biochemical analyses

Serum total cholesterol (TC), triglycerides (TG), glutamic pyruvic transaminase (GPT), glutamic oxaloacetic transaminase (GOT), and high-density lipoprotein cholesterol (HDL) were measured colorimetrically using commercial assay kits from Asan Pharmaceutical (Seoul, Republic of Korea), as described previously (Yun et al., 2024). Serum insulin and HbA1c were determined by ELISA kit (Morinaga, Yokohama, Japan; MyBioSource Inc., CA, United States).

### 2.8 Histology

Liver and epididymal fat tissue samples from mice were fixed in 4% formalin and embedded in paraffin (FFPE, formalin-fixed and paraffin-embedded). The tissues were sectioned at a 4 µm thickness using a rotary microtome (RM2235; Leica, Nussloch, Germany). Sections were deparaffinized and rehydrated via serial passages through xylene and graded ethanol for subsequent hematoxylin and eosin (H&E) staining, as described previously (Choi et al., 2021). Images were acquired using a light microscope (BX53; Olympus) equipped with a digital camera under identical illumination and exposure settings for all experimental groups. Images were captured at a fixed magnification.

Adipocyte size in epididymal adipose tissue sections was quantified using ImageJ software as previous described (Hu et al., 2021). Briefly, H&E-stained images were converted to 8-bit grayscale, and adipocytes were segmented using an automatic thresholding method (Otsu’s algorithm). Individual adipocytes were identified using the “Analyze Particles” function after watershed separation to distinguish adjacent cells. Cells that were incomplete, distorted, or located at the image borders were excluded from the analysis. For each mouse, adipocyte size was quantified from five randomly selected microscopic fields, and at least 100 adipocytes were analyzed per section. The mean adipocyte area was calculated and used for statistical analysis.

### 2.9 Protein extraction and Western blot analysis

Liver tissues (50–100 mg) were homogenized in EzRIPA lysis buffer (WSE-7420; ATTO, Tokyo, Japan) supplemented with 1% Xpert duo inhibitor cocktail solution (GenDEPOT, TX, United States). The homogenates were centrifuged at 12,000x *g* for 10 min at 4 °C, and the supernatant was collected. Total protein concentrations were determined using a Bradford assay (Bio-Rad, Hercules, CA, United States) according to the manufacturer’s instructions.

Equal amounts of protein were mixed with Laemmli sample buffer (Bio-Rad, Hercules, CA, United States) containing 5% β-mercaptoethanol, and denatured by heating at 95 °C for 5 min. Proteins were separated by SDS–polyacrylamide gel electrophoresis (SDS–PAGE) and transferred onto polyvinylidene difluoride (PVDF) membranes (IPVH00010; Millipore, Billerica, MA, United States) using a Mini Trans-Blot^®^ electrophoretic transfer system (Bio-Rad).

Membranes were blocked for 1 h at room temperature in Tris-buffered saline containing 0.1% Tween 20 (TBST) and 5% skim milk (Becton Dickinson, Sparks, MD, United States). After washing with TBST, membranes were incubated overnight at 4 °C in TBST containing 5% bovine serum albumin (MP Biomedicals, Irvine, CA, United States) with primary antibodies against phosphorylated AKT (p-AKT), total AKT, phosphorylated AMPK (p-AMPK), and total AMPK (Cell Signaling Technology, Danvers, MA, United States). Following primary antibody incubation, membranes were washed with TBST and incubated for 1 h at room temperature with horseradish peroxidase (HRP)-conjugated anti-mouse or anti-rabbit IgG secondary antibodies (Santa Cruz Biotechnology, Dallas, TX, United States), as appropriate. Immunoreactive protein bands were visualized using enhanced chemiluminescence (ECL) reagent (34075; Thermo Scientific, Waltham, MA, United States) and detected with a ChemiDoc XRS imaging system (Bio-Rad).

Densitometric analysis was performed using ImageJ software after background subtraction. Phosphorylated AKT and AMPK levels were quantified by normalization to their corresponding total AKT or total AMPK levels (p-AKT/AKT and p-AMPK/AMPK) to assess pathway activation. All densitometric analyses were conducted using identical settings across all samples.

### 2.10 *C. elegans* culture and treatment

Wild-type N2 *C. elegans* (Bristol) strain and *Escherichia coli* (*E. coli*) OP50 were obtained from the *C. elegans* and Nematode Bank (CeNBank, Republic of Korea) and maintained on nematode growth medium (NGM) at 20 °C. The High-Glucose Diet (HGD) *C. elegans* model was established by exposing N2 worms to NGM supplemented with 2% glucose (Alcántar-Fernández et al., 2018). Age synchronization was achieved by treating worms with hypochlorite to dissolve the adult bodies and isolate the eggs. The collected eggs were cultured at 20 °C until they reached the L4 stage, either in the presence or absence of glucose. Experimental groups included a normal control group without glucose, a high-glucose group (2% glucose), treatment groups exposed to 2% glucose supplemented with GGT (0.5, 1.0, or 2.0 mg/mL), and a metformin-treated group (50 mM) under high-glucose conditions, which served as a pharmacological reference control (Mejia-Martinez et al., 2017). Synchronized L1-stage worms were grown to the L4 stage in media supplemented with the respective compounds to assess body size. Worm images were acquired using an Olympus BX53 microscope equipped with a digital camera and cellSens acquisition software under identical imaging settings for all groups. Images were captured at a fixed magnification and analyzed using ImageJ software.

### 2.11 Lifespan assay in *C. elegans*

After synchronization, L4 stage worms were cultured on NGM plates containing 50 μM floxuridine (FUDR) for 3 days. Fifty L4 stage worms were transferred to plates containing the respective compounds, and the number of dead worms was counted every 48 h. Surviving worms were transferred to fresh NGM plates every 2 days. Worms were considered dead if they did not respond to gentle probing with a platinum wire. Survival data were analyzed by generating Kaplan–Meier curves.

### 2.12 Lipid staining and quantification in *C. elegans*

Oil Red O (ORO; Sigma-Aldrich) and Nile Red (NR; Sigma-Aldrich) staining methods, with minor modifications based on a previously described protocol (Escorcia et al., 2018). Fresh ORO staining solution was prepared by diluting ORO stock solution (0.5% in isopropanol) with distilled water at a 6:4 ratio, followed by filtration. To prepare the NR working solution, 5 mg/mL NR stock was dissolved in 100% acetone. L4 stage worms were collected, washed, and fixed in 60% or 40% isopropanol for 3 min. After removing isopropanol, worms were stained with the prepared ORO or NR solution for 2 h. Following staining, worms were washed with phosphate-buffered saline containing 0.01% Triton X-100 (PBST) to remove excess dye. For ORO staining, worms were mounted on microscope slides (HSU-0810001; Marienfeld Superior, Germany) and observed using an Olympus BX53 Microscope. For NR staining, worms were observed using a fluorescence microscope (Olympus U-RFL-T) attached to an Olympus BX53 Microscope. All images were captured using identical microscope settings, including exposure time and gain, within each staining modality. Quantification of ORO and NR fluorescence intensity was performed using ImageJ by measuring mean pixel intensity after background subtraction. At least 30 worms per group were analyzed, and all image analysis parameters were kept constant across groups.

Triglyceride (TG) levels were measured using the PicoSens™ TG Assay Kit (Biomax, Republic of Korea), following the manufacturer’s protocol. Absorbance was read at 570 nm using a Tecan Spark 10M Multimode Plate Reader with SPARKCONTROL software (Version 2.1).

### 2.13 Glucose uptake assay in *C. elegans*

After synchronization, *C. elegans* at the L4 or adult-stage were collected and incubated at 20 °C for 2 h in M9 buffer containing 0.5 mM 2-deoxyglucose (2-DG) (provided with the kit). Glucose uptake was assessed using a commercial assay kit (ab136955; Abcam, United Kingdom), following the manufacturer’s instructions. The absorbance was measured at 412 nm using a TECAN Spark 10M Multimode Plate Reader and SPARKCONTROL software.

### 2.14 Quantitative Real-time PCR (qPCR)

Quantitative Real-time PCR was performed using the TRIzol reagent kit (Invitrogen) according to the manufacturer’s protocol. For qPCR analysis, SYBR^®^ Green Real-time PCR Master Mix (TOYOBO, Japan) using the LightCycler^®^ 96 platform (Roche, Germany). The ΔC_t_ value was determined as the difference between the Ct values of the target and housekeeping genes. Relative gene expression was quantified using the 2^−ΔΔCT^ method (Livak and Schmittgen, 2001), where ΔΔC_t_ represents the difference between the ΔC_t_ values of the experimental and control groups. Data was analyzed using LightCycler software (version 1.1; Roche Applied Science). The primer sequences are listed in Supplementary Table S3.

### 2.15 Statistical analysis

Data from individual experiments are presented as mean ± SD. Group comparisons were made using one-way ANOVA, followed by Dunnett’s multiple comparison test to assess the effects of the treatments. Statistical analyses were performed using GraphPad Prism 8.0.1 (GraphPad Software, San Diego, CA, United States). Statistical significance was set at *p* < 0.05.

## 3 Results

### 3.1 Effects of GGT on glucose uptake and gene expression in C2C12 myotubes

**FIGURE 1:**
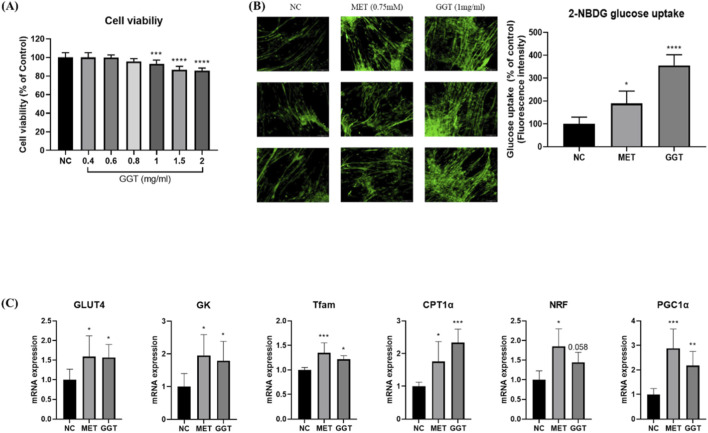
Effects of Galgeun-tang (GGT) on cell viability, glucose uptake, and metabolic gene expression in C2C12 myotubes. **(A)** Cell viability after 24 h treatment with increasing concentrations of GGT (0.4–2.0 mg/mL). **(B)** Glucose uptake measured by 2-NBDG fluorescence following treatment with metformin (MET; 0.75 mM) or GGT (1.0 mg/mL). **(C)** mRNA expression of GLUT4, GK, Tfam, CPT1α, NRF, and PGC1α determined by qPCR. Data are presented as mean ± SD. Statistical significance was determined by one-way ANOVA followed by Dunnett’s test. *p < 0.05, **p < 0.01, ***p < 0.001, ****p < 0.0001 vs. NC group.

The effect of GGT on cell viability was also analyzed. Compared to the NC group, 0.4, 0.6, and 0.8 mg/mL GGT did not induce a statistically significant difference in cell viability. However, the 1, 1.5, and 2 mg/mL GGT groups showed a significant reduction in cell viability compared to the NC group (*p <* 0.001*, p <* 0.0001, and *p <* 0.0001, respectively) (Figure 1A). In differentiated myotubes, both GGT and MET groups significantly enhanced glucose uptake compared with the NC group (*p* < 0.05 and *p* < 0.0001, respectively) (Figure 1B). After qPCR analysis in C2C12 myotubes, the MET and GGT groups showed significant upregulation of GLUT4, GK, Tfam, CPT1α, and PGC1α, while NRF expression was significantly increased only by MET (Figure 1C).

### 3.2 Effects of GGT supplementation in HFD-induced obese mice

#### 3.2.1 Body weight and food intake

**FIGURE 2:**
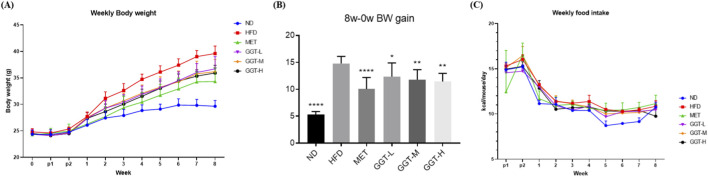
Effects of GGT on body weight gain and food intake in HFD-induced obese mice. **(A)** Weekly changes in body weight during the 8-week experimental period. **(B)** Total body weight gain after 8 weeks **(C)** Average weekly food intake. ND, normal diet; HFD, high-fat diet; MET, metformin. Data are presented as mean ± SD (n = 8). Statistical analysis by one-way ANOVA with Dunnett’s test. *p < 0.05, **p < 0.01, ***p < 0.001, ****p < 0.0001 vs. HFD group.

Eight weeks of HFD feeding increased the body weight gain. The MET group exhibited the most pronounced reduction in weight gain, and all three GGT-treated groups (GGT-L, GGT-M, and GGT-H) significantly suppressed weight gain compared with the HFD group (Figures 2A,B).

Food intake was monitored weekly, beginning 2 weeks before the study initiation and continuing for 8 weeks. Although no statistically significant differences were observed, the ND group exhibited the lowest food intakes from weeks 3–7. However, at 2 and 8 weeks, the GGT-H group showed the lowest food intake among all the groups, with no statistical significance (Figure 2C).

#### 3.2.2 Glucose tolerance and fasting glucose

**FIGURE 3:**
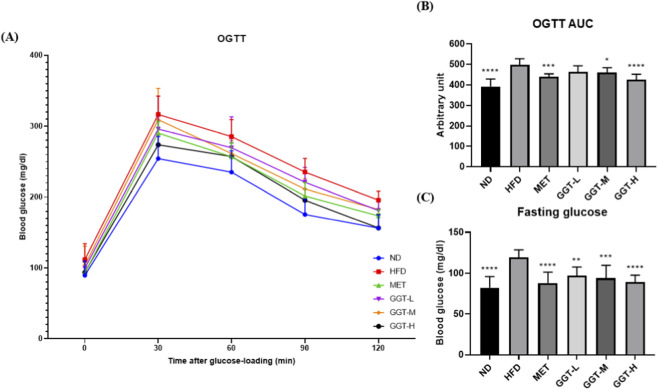
Effects of GGT on glucose tolerance and fasting glucose in HFD-fed mice. **(A)** OGTT blood glucose profiles measured at 0, 30, 60, 90, and 120 min after glucose loading (2 g/kg). **(B)** OGTT area under the curve (AUC). **(C)** Fasting blood glucose levels after overnight fasting. Data are presented as mean ± SD (n = 8). Statistical analysis by one-way ANOVA with Dunnett’s test. *p < 0.05, **p < 0.01, ***p < 0.001, ****p < 0.0001 vs. HFD group.

OGTT measurements showed that MET and GGT supplementation improved glucose tolerance, with GGT-H exhibiting values comparable to ND between 30 and 120 min (Figure 3A). The OGTT area under the curve (AUC) was significantly reduced in MET, GGT-M, and GGT-H groups (Figure 3B). Fasting glucose levels were significantly lower in all treatment groups compared with HFD (Figure 3C).

#### 3.2.3 Organ weights and histological analysis

**FIGURE 4:**
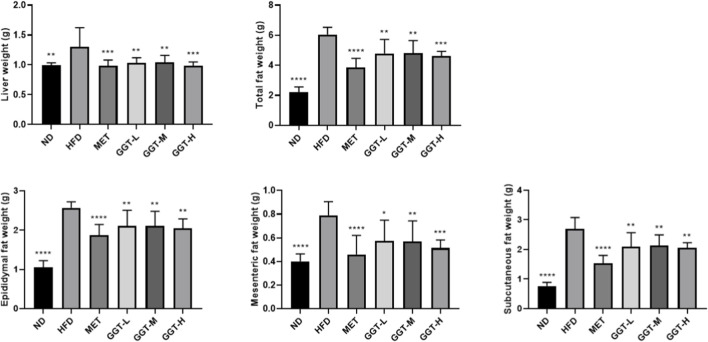
Effects of GGT on liver and adipose tissue weights in HFD-fed mice. Liver weight, total fat weight, and individual adipose depots (epididymal, mesenteric, subcutaneous). Data are presented as mean ± SD (n = 8). Statistical analysis by one-way ANOVA with Dunnett’s test. *p < 0.05, **p < 0.01, ***p < 0.001, ****p < 0.0001 vs. HFD group.

The weights of the liver, total fat, epididymal fat, mesenteric fat, and subcutaneous fat were measured. HFD feeding increased liver and adipose tissue weights. MET and all GGT doses significantly reduced liver, total fat, and adipose depot weights, with GGT-H showing the largest reduction in total and mesenteric fat (Figure 4). Histological examination revealed extensive hepatic and adipose lipid accumulation in HFD-fed mice, which was markedly attenuated by MET, GGT-M, and GGT-H (Supplementary Figure S1).

#### 3.2.4 Serum biochemical parameters

**FIGURE 5:**
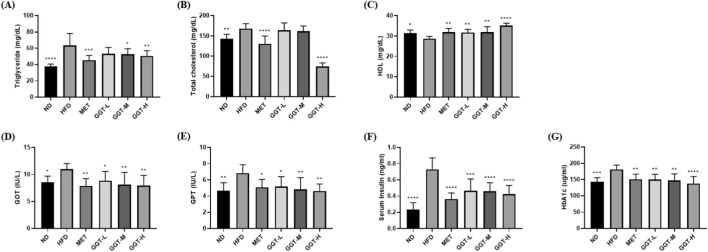
Effects of GGT on serum lipid profiles, liver enzymes, and glycemic parameters in HFD-fed mice. **(A)** Triglycerides (TG), **(B)** total cholesterol (TC), **(C)** high-density lipoprotein cholesterol (HDL), **(D)** glutamic oxaloacetic transaminase (GOT), **(E)** glutamic pyruvic transaminase (GPT), **(F)** serum insulin, and **(G)** HbA1c levels. Data are presented as mean ± SD (n = 8). Statistical analysis by one-way ANOVA with Dunnett’s test. *p < 0.05, **p < 0.01, ***p < 0.001, ****p < 0.0001 vs. HFD group.

Compared to the HFD group, the MET and GGT-H groups exhibited significant reductions in serum TG and TC levels (Figures 5A,B). In addition, HDL levels significantly increased in all treatment groups, with GGT-H showing a significantly greater difference (Figure 5C). Regarding liver function, the MET and GGT groups showed significantly decreased GOT and GPT levels compared with those in the HFD group (Figures 5D,E). Regarding glucose metabolism, the MET and all three GGT-supplemented groups exhibited significantly lower serum insulin and HbA1c levels than the HFD group, indicating improved glycemic regulation (Figures 5F,G).

#### 3.2.5 Hepatic and adipose gene expression

In the liver, MET and all GGT groups downregulated lipogenesis-related genes (FAS, PPARγ, ACC1, SCD1, and C/EBPα) and upregulated β-oxidation markers (PPARα and PGC1α). Gluconeogenic genes (PEPCK, G6Pase, and GLUT2) were suppressed, with the most pronounced effects in GGT-H. Pro-inflammatory cytokines (TNFα and IL-6) were significantly reduced, particularly IL-6 in all GGT groups (Supplementary Figure S2).

In adipose tissue, the MET and GGT-H groups significantly suppressed the expression of PPARγ, SREBP1c, ACC1, and LPL compared to the HFD group. The PPARα expression levels in the GGT-L, GGT-M, and GGT-H groups were higher than those in the HFD group, although the difference was not statistically significant. Conversely, the GGT-M and GGT-H groups demonstrated a statistically significant upregulation of PGC1α expression compared to that in the HFD group (Supplementary Figure S3).

#### 3.2.6 Hepatic protein expression

Hepatic AKT and AMPK signaling pathways were examined by Western blot analysis (Supplementary Figure S4). When normalized to their corresponding total protein levels, the p-AKT/AKT ratio was significantly increased in the GGT-L group compared with the HFD group, indicating enhanced AKT activation at the low dose of GGT. No statistically significant changes in the p-AKT/AKT ratio were observed in the GGT-M or GGT-H groups relative to the HFD group.

For AMPK signaling, no statistically significant differences were detected in the p-AMPK/AMPK ratio among experimental groups. Although treatment groups exhibited lower mean p-AMPK/AMPK values compared with the HFD group, these differences did not reach statistical significance.

### 3.3 Effects of GGT in *C. elegans*

#### 3.3.1 Growth and lifespan

**FIGURE 6:**
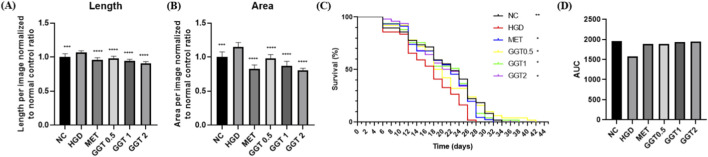
Effects of GGT on body size and lifespan in high-glucose diet (HGD)-fed *C*. *elegans*. **(A)** Body length, **(B)** body width, and **(C)** survival curves of worms treated with MET or GGT (0.5, 1.0, or 2.0 mg/mL) under HGD conditions. Data are presented as mean ± SD. Statistical analysis by one-way ANOVA with Dunnett’s test for body size and log-rank test for survival curves. **(D)** Area under the curve (AUC) of *C. elegans* survival curves. *p < 0.05, **p < 0.01, ***p < 0.001, ****p < 0.0001 vs. HGD group.

In the high-glucose diet (HGD) model, worm body length and width were increased compared with NC, whereas MET and all GGT doses significantly reduced both parameters (Figures 6A,B). Survival analysis showed improved lifespan in MET and GGT-treated groups, with GGT 0.5 mg/mL showing the highest survival rate (Figures 6C,D).

#### 3.3.2 Lipid accumulation and glucose uptake

**FIGURE 7:**
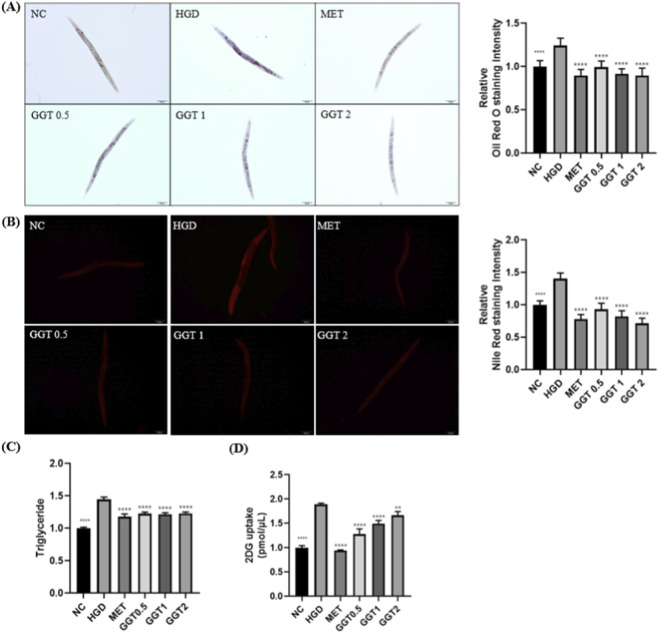
Effects of GGT on lipid accumulation, triglyceride content, and glucose uptake in HGD-fed *C. elegans*. Representative images and quantification from **(A)** Oil Red O (ORO) and **(B)** Nile Red (NR) staining, **(C)** triglyceride assay, and **(D)** glucose uptake assay. Data are presented as mean ± SD. Statistical analysis by one-way ANOVA with Dunnett’s test for body size and log-rank test for survival curves. *p < 0.05, **p < 0.01, ***p < 0.001, ****p < 0.0001 vs. HGD group.

The effects of GGT on lipid accumulation and glucose uptake in HGD-induced *C. elegans* were evaluated using ORO and NR staining, TG quantification, and glucose uptake assays. ORO and Nile Red staining revealed that lipid accumulation induced by HGD was reduced by MET and all GGT treatments (Figures 7A,B). Triglyceride levels and glucose uptake were also decreased, indicating improved metabolic regulation (Figures 7C,D).

#### 3.3.3 Gene expression

The effects of GGT on lipid metabolism–and insulin signaling–related gene expression were evaluated in *C. elegans* exposed to a high-glucose diet (HGD). Under HGD conditions, the expression of several lipogenesis-associated genes, including *fat-4*, *fat-6*, and *fat-7*, was significantly increased compared with the normal control. Treatment with metformin and GGT partially attenuated the expression of these genes, although the magnitude of the response varied depending on the dose. In contrast, *fat-5* expression exhibited a distinct pattern and remained elevated relative to the normal control across treatment groups, indicating gene-specific regulatory responses under high-glucose conditions (Supplementary Figure S5A).

For lipolysis-related genes (*mdt-15*, *nhr-76*, and *atgl-1*), distinct expression patterns were observed under high-glucose conditions. Compared with the NC, *mdt-15* expression was markedly downregulated and *nhr-76* expression was upregulated in the HGD group, whereas *atgl-1* expression was not significantly altered. Relative to the HGD group, treatment with MET and GGT at 0.5 mg/mL significantly increased *mdt-15* expression, while *nhr-76* expression was significantly elevated only in the GGT 2.0 mg/mL group. *Atgl-1* expression remained unchanged across all treatment groups (Supplementary Figure S5B).

For fatty acid β-oxidation–related genes (*nhr-80*, *cpt-1*, and *nhr-49*), expression levels were generally higher under HGD conditions than in the normal control. Relative to the HGD group, *nhr-80* expression was significantly increased by GGT at 0.5 and 2.0 mg/mL, whereas the GGT 1.0 mg/mL did not reach statistical significance. *Cpt-1* expression was significantly upregulated only in the GGT 2.0 mg/mL group. *Nhr-49* showed a modest, non-significant upward trend at 0.5 mg/mL (Supplementary Figure S5C).

In the insulin/IGF-1 signaling pathway, *daf-16* expression was reduced, and *daf-2* expression was increased in the HGD group compared with the NC. Relative to the HGD group, treatment with MET, GGT 0.5 mg/mL, and GGT 2.0 mg/mL significantly increased *daf-16* expression, while *daf-2* expression was significantly decreased by MET, GGT 0.5 mg/mL, and GGT 1.0 mg/mL (Supplementary Figure S5D).

## 4 Discussion

This study demonstrates that *Galgeun-tang* (GGT) exerts anti-obesity effects through coordinated regulation of lipid, glucose, and energy metabolism across cellular, organismal, and systemic experimental models. While prior investigations have primarily examined the anti-obesity effects of GGT as a formula in comparison with high-fat diet conditions or conventional pharmacological agents, the present work provides a comprehensive evaluation of the dose-dependent actions of the full formula across C2C12 myotubes, *C. elegans* (*C. elegans*), and high-fat diet (HFD)-induced obese mice (Jeong et al., 2014). Similar anti-obesity trends of GGT have also been reported in regional studies using HFD-fed mice (Ki et al., 2016; Ye et al., 2016).

The rationale for employing this tiered experimental design was to capture complementary aspects of metabolic regulation that cannot be fully addressed by a single model. C2C12 myotubes allowed direct assessment of muscle cell–specific glucose uptake and mitochondrial responses under controlled conditions. *C. elegans* was incorporated as a supportive whole-organism *in vivo* model in which evolutionarily conserved metabolic processes related to lipid metabolism, insulin/IGF-1 signaling, energy balance, and lifespan can be assessed at the organismal level. This model enables rapid assessment of systemic metabolic responses and gene regulatory networks under high-glucose stress, thereby bridging the gap between cellular assays and mammalian physiology. The HFD-induced mouse model was used to validate these findings in a complex mammalian system that recapitulates key features of human obesity and metabolic dysfunction.

GGT consistently inhibited lipogenesis and promoted lipolysis across models. In HFD-induced obese mice, hepatic and adipose lipid accumulation was markedly reduced, serum lipid profiles improved, and histological steatosis was alleviated. These changes were accompanied by downregulation of lipogenesis-associated genes and upregulation of β-oxidation markers. Although multiple adipose depots were collected, histological and gene-expression analyses focused on epididymal adipose tissue, a well-established visceral fat depot that is highly responsive to diet-induced metabolic stress and closely associated with systemic insulin resistance and inflammation in rodents (He et al., 2020; Hu et al., 2021). While this approach provides a robust and commonly used index of obesity-related metabolic dysfunction, depot-specific effects of GGT in mesenteric and subcutaneous adipose tissues warrant further investigation.

In *C. elegans*, GGT supplementation suppressed lipid accumulation, as demonstrated by Oil Red O and Nile Red staining, and significantly reduced triglyceride levels. Gene expression analyses revealed consistent downregulation of lipogenesis-associated genes, whereas genes related to lipolysis and fatty acid β-oxidation exhibited mixed patterns. Notably, *nhr-80* and *cpt-1* were upregulated at specific GGT concentrations, while *nhr-49* and *atgl-1* showed limited or non-significant responses. These non-linear transcriptional patterns likely reflect dose-dependent engagement of distinct regulatory pathways, saturation effects, or compensatory feedback mechanisms that are characteristic of whole-organism metabolic regulation under high-glucose conditions.

The lipid-lowering effects observed in this study are supported by previous reports demonstrating the anti-adipogenic and metabolic regulatory properties of major metabolites of GGT. Bioactive plant metabolites such as puerarin have been shown to inhibit adipocyte differentiation and improve lipid metabolism (Chen et al., 2018; Buhlmann et al., 2019), while *E. sinica* and its metabolites promote energy expenditure and reduce lipid accumulation in obesity models (Kim et al., 2014; Song et al., 2022). These metabolite-level effects provide a mechanistic basis for the lipid-lowering actions of the GGT formula observed in this study and are consistent with earlier studies reporting anti-obesity effects of GGT in HFD-induced obese mice (Jeong et al., 2014), with comparable observations reported in regional studies (Ki et al., 2016; Ye et al., 2016).

GGT supplementation also enhanced energy metabolism and mitochondrial activity. In C2C12 myotubes, GGT increased the expression of mitochondrial biogenesis markers and fatty acid transport–related genes, indicating enhanced oxidative capacity. In HFD-fed mice, GGT reduced body weight gain and increased hepatic expression of PPARα and PGC1α, suggesting activation of β-oxidation pathways. Although *in vivo* skeletal muscle analyses were not performed in the present study, the C2C12 findings provide mechanistic evidence that muscle tissue may contribute to the systemic metabolic improvements observed. Future studies incorporating *in vivo* muscle-specific analyses will be required to more fully define the role of skeletal muscle in mediating the metabolic effects of GGT.

Glucose homeostasis was consistently improved across models. In C2C12 myotubes, GGT enhanced glucose uptake and upregulated GLUT4 and glucokinase expression. In HFD-fed mice, fasting glucose, insulin, HbA1c, and OGTT responses were significantly improved by GGT treatment, accompanied by suppression of gluconeogenic gene expression. Hepatic AKT phosphorylation was significantly enhanced at the low dose of GGT, whereas higher doses did not elicit a statistically significant increase, indicating a dose- and context-dependent modulation of insulin signaling. Such non-linear dose–response patterns have been reported for multi-component formulations and may reflect saturation of insulin signaling pathways, activation of compensatory feedback mechanisms, or differential engagement of bioactive metabolites at higher doses.

In *C. elegans*, GGT was associated with altered expression of insulin signaling–related genes, including *daf-2* and *daf-16*, supporting the organismal-level metabolic effects observed across models. Collectively, these findings are consistent with previous studies indicating that GGT and its major metabolites regulate lipid metabolism and glucose homeostasis and are generally well tolerated in preclinical and clinical contexts (Buhlmann et al., 2019; Yang et al., 2019; Yoo et al., 2021).

From a clinical perspective, the significance of this study lies in the exploration of GGT as a multi-targeted therapeutic option for obesity and metabolic dysfunction. Although glucagon-like peptide-1 (GLP-1) receptor agonists and dual GLP-1/GIP receptor agonists have shown substantial efficacy in weight loss, their clinical use remains controversial due to gastrointestinal adverse effects, long-term tolerability concerns, and high discontinuation rates. In this context, traditional multi-component formulation such as GGT offers a complementary approach with a distinct mechanism of action. Rather than acting through a single hormonal pathway, GGT demonstrated coordinated regulation of lipid metabolism, glucose homeostasis, insulin signaling, and energy expenditure across multiple biological levels in the present study. This multi-pathway modulation may provide metabolic benefits while potentially reducing the risk of severe adverse effects associated with mono-target pharmacotherapies. Although further clinical studies are required to establish efficacy, safety, and optimal dosing, the present findings provide a preclinical rationale supporting the potential clinical application of GGT as an adjunct or alternative strategy for obesity management.

## 5 Conclusion

In conclusion, GGT exerts multi-targeted anti-obesity effects by modulating lipid metabolism, glucose homeostasis, insulin signaling, and energy expenditure across cellular, organismal, and mammalian models. Using a tiered experimental approach, this study demonstrates that GGT suppresses lipogenesis, enhances fatty acid oxidation, improves insulin sensitivity, and promotes mitochondrial activity, while exhibiting dose- and context-dependent regulatory patterns characteristic of multi-component formulations. The complementary findings obtained from C2C12 myotubes and HFD–induced obese mice provide mechanistic insight into the metabolic actions of GGT, while the *C. elegans* data offer supportive organismal-level evidence. Although further studies are required to define tissue-specific mechanisms, optimal dosing, long-term efficacy, and clinical safety, the present results offer a robust preclinical rationale supporting the potential application of GGT as an adjunct or alternative strategy for the management of obesity and metabolic dysfunction.

## Data Availability

The raw data supporting the conclusions of this article will be made available by the authors, without undue reservation.

## Supplementary material

SUPPLEMENTARY FIGURE S1 Histological analysis of liver and adipose tissues in HFD-fed mice. Representative H&E-stained sections of **(A)** liver and **(B)** epididymal adipose tissue from each group (×200, scale bar: 100 μm).

SUPPLEMENTARY FIGURE S2 Hepatic gene expression in HFD-fed mice. mRNA levels of genes related to lipogenesis, β-oxidation, gluconeogenesis, and inflammation in liver tissues. Data are presented as mean ± SD (n = 8). Statistical analysis by one-way ANOVA with Dunnett’s test. **p* < 0.05, ***p* < 0.01, ****p* < 0.001, *****p* < 0.0001 vs. HFD group.

SUPPLEMENTARY FIGURE S3 Adipose tissue gene expression in HFD-fed mice. mRNA levels of genes related to adipogenesis, lipogenesis, and β-oxidation in epididymal adipose tissue. Data are presented as mean ± SD (n = 8). Statistical analysis by one-way ANOVA with Dunnett’s test. **p* < 0.05, ***p* < 0.01, ****p* < 0.001, *****p* < 0.0001 vs. HFD group.

SUPPLEMENTARY FIGURE S4 Effects of GGT on hepatic AKT and AMPK signaling pathways in HFD-fed mice. Representative Western blot images show phosphorylated and corresponding total forms of AKT and AMPK in liver tissues from mice fed a normal diet (ND), high-fat diet (HFD), treated with metformin (MET), or treated with GGT (GGT-L, GGT-M, and GGT-H). **(A)** Representative blots of p-AKT, total AKT, p-AMPK, and total AMPK. **(B)** Densitometric quantification of p-AKT and p-AMPK normalized to their corresponding total AKT or total AMPK levels (p-AKT/AKT and p-AMPK/AMPK). Densitometric analyses were performed using ImageJ software. Phosphorylated protein levels were quantified relative to the corresponding total kinase levels to assess pathway activation. Data are presented as mean ± SD. Statistical significance was determined relative to the HFD group (**p* < 0.05, ***p* < 0.01).

SUPPLEMENTARY FIGURE S5 Gene expression in HGD-fed *C. elegans*. **(A)** Lipogenesis-related genes, **(B)** lipolysis-related genes, **(C)** β-oxidation–related genes, and **(D)** insulin signaling–related genes. Data are presented as mean ± SD (n = 3). Statistical analysis by one-way ANOVA with Dunnett’s test. **p* < 0.05, ***p* < 0.01, ****p* < 0.001, *****p* < 0.0001 vs. HGD group.
