# Supplementary figures and images for: Galgeun-tang modulates lipid, glucose, and energy metabolism in diet-induced obesity across cellular, nematode, and murine models
Source: Front Pharmacol. 2026 Feb 27;17:1747882. doi: 10.3389/fphar.2026.1747882 (PMC12982924; doi:10.3389/fphar.2026.1747882)

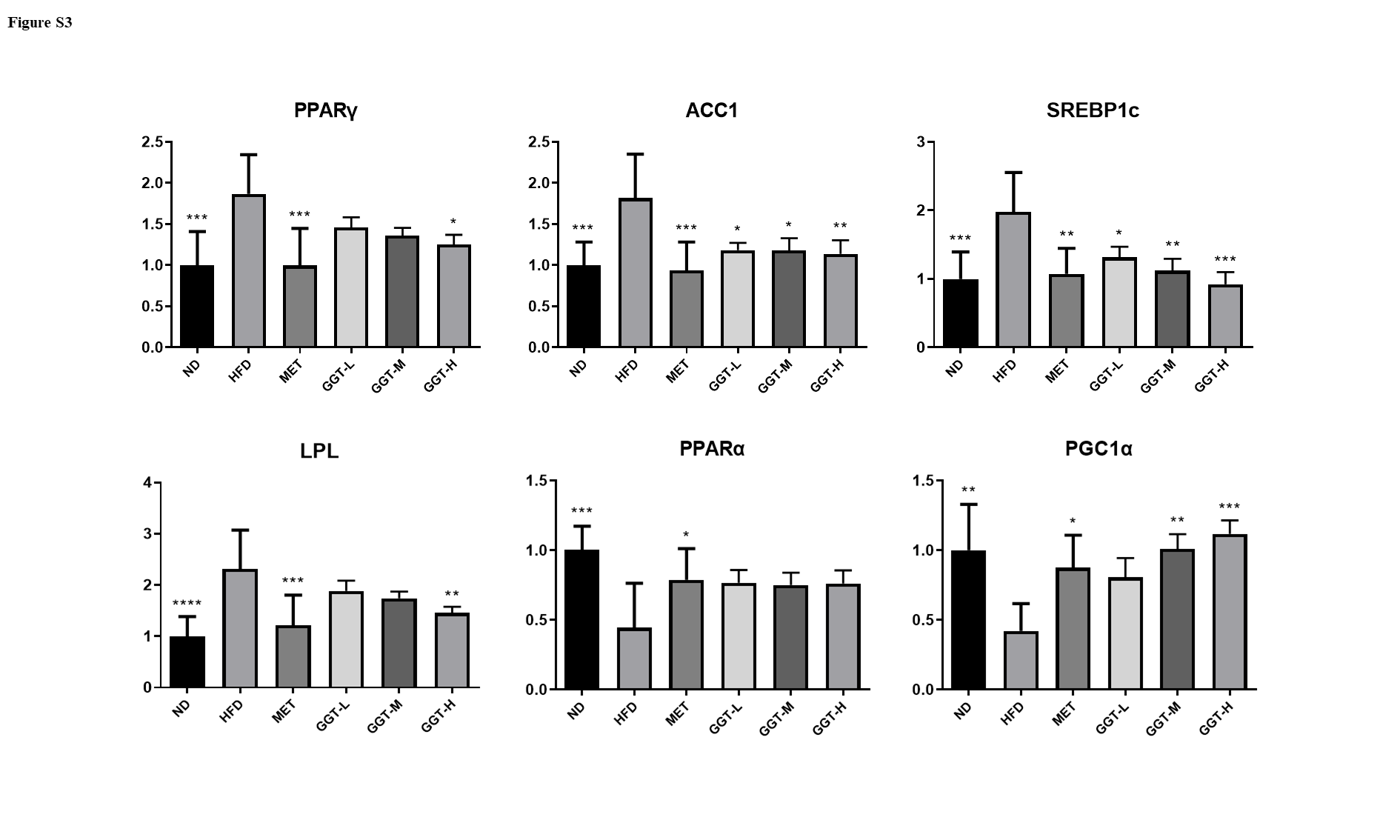

Supplement: Supplementary file 1 [file Image3.tif]

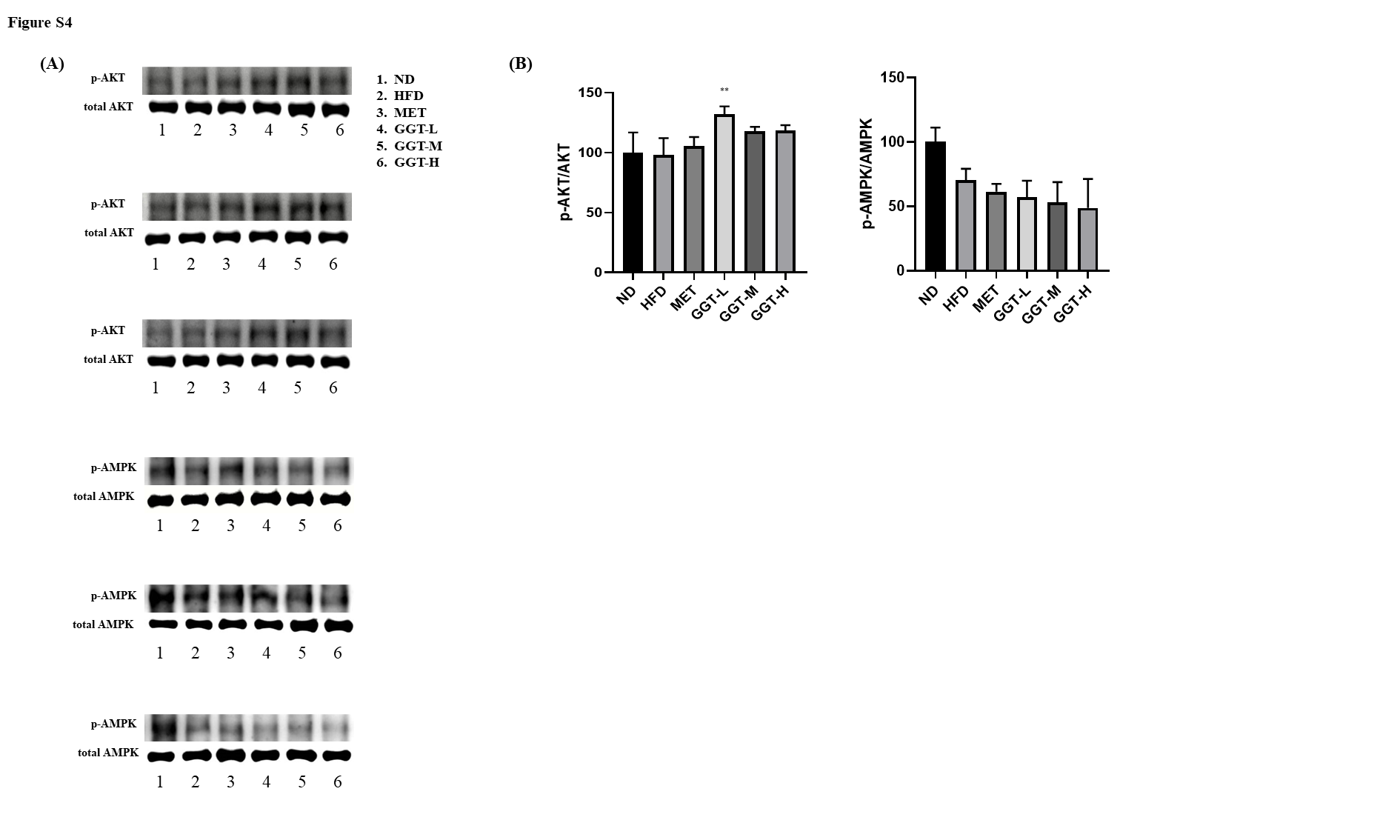

Supplement: Supplementary file 2 [file Image4.tif]

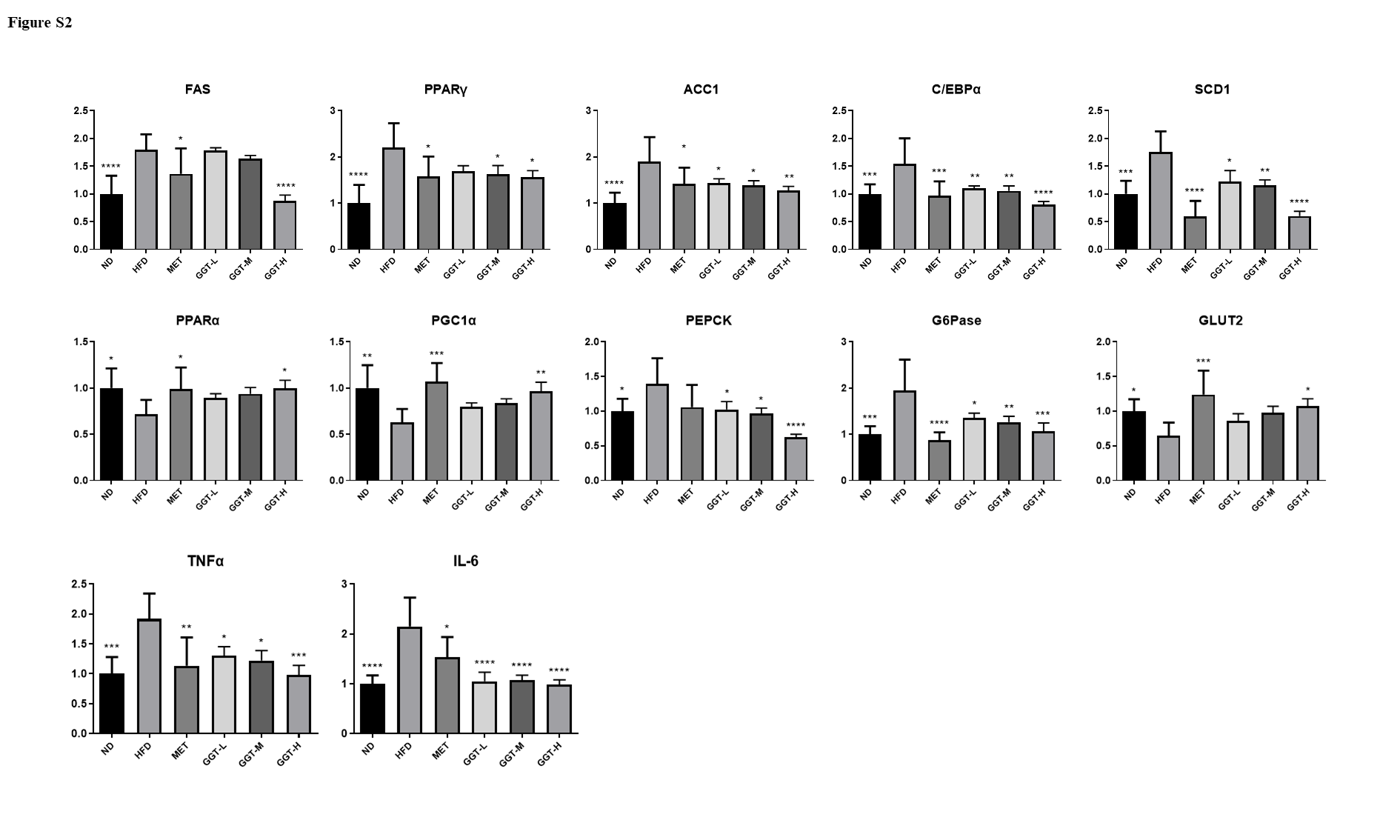

Supplement: Supplementary file 3 [file Image2.tif]

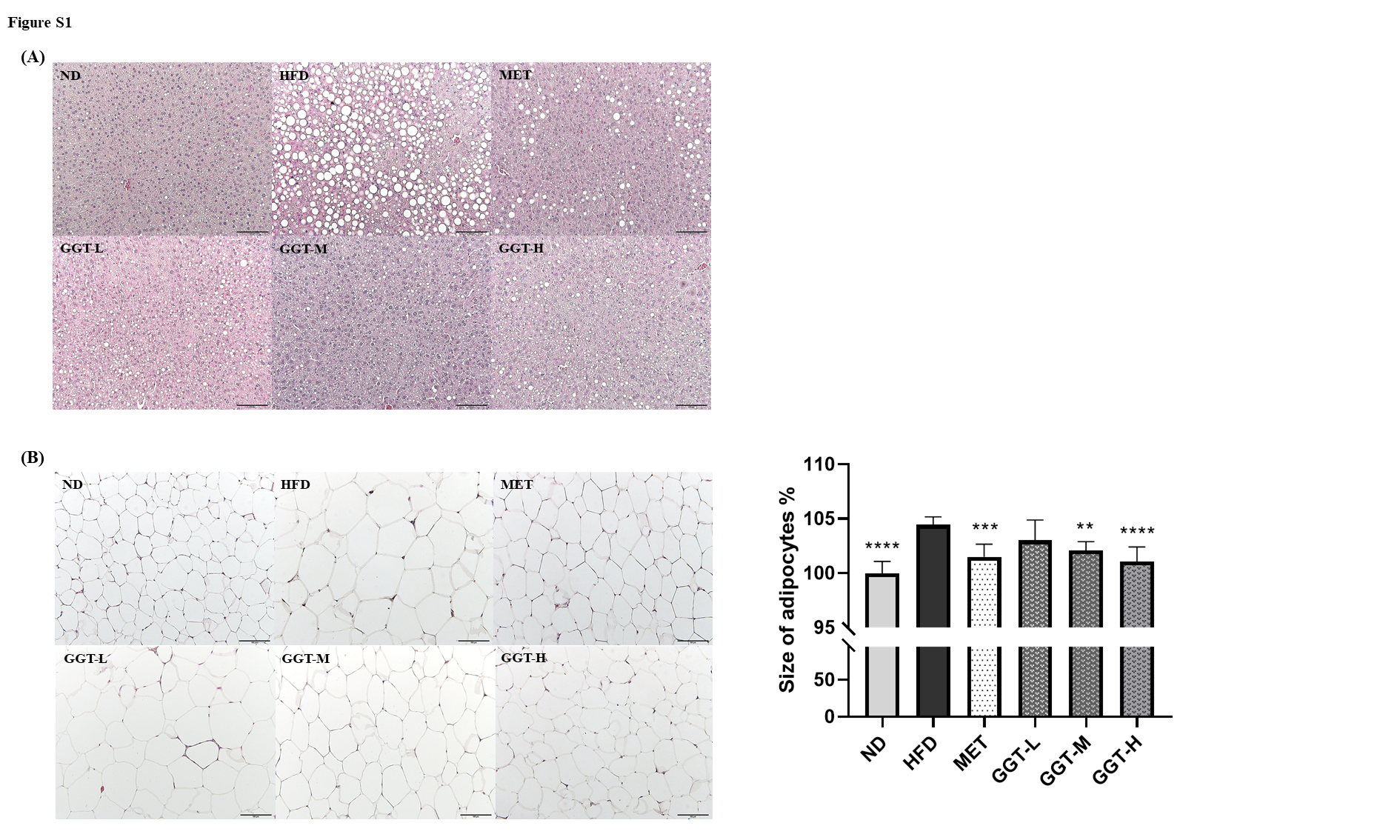

Supplement: Supplementary file 4 [file Image1.tif]

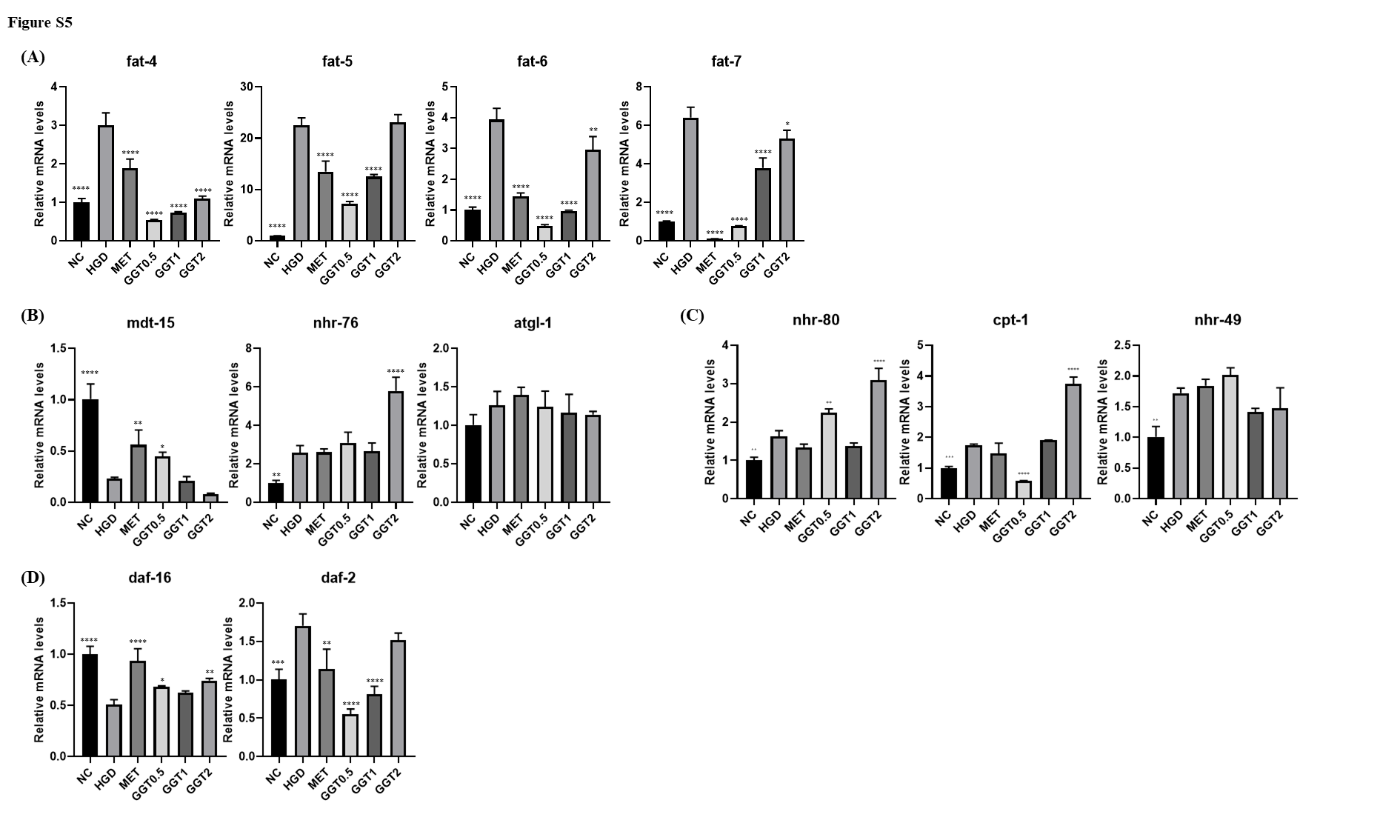

Supplement: Supplementary file 5 [file Image5.tif]
